# Norrin Ameliorates Retinal Ganglion Cell Apoptosis by Normalizing VEGF and PEDF Dysregulation in Diabetic Retinopathy

**DOI:** 10.3390/cells15080689

**Published:** 2026-04-14

**Authors:** Chan-Hee Moon, Tae-Yong Koh, Ji-Seok Yoon, Minsoo Kim, Kwon-Soo Ha

**Affiliations:** 1 Department of Molecular and Cellular Biochemistry, Kangwon National University School of Medicine, Chuncheon 24341, Kangwon-do, Republic of Korea; mch0419@knuh.or.kr (C.-H.M.); 202416126@kangwon.ac.kr (T.-Y.K.); jisuk1027@kangwon.ac.kr (J.-S.Y.); 2 Department of Anesthesiology, Kangwon National University School of Medicine, Chuncheon 24341, Kangwon-do, Republic of Korea; kmsanp@kangwon.ac.kr

**Keywords:** diabetic retinopathy, neurodegeneration, norrin, pigment epithelium-derived factor, vascular endothelial growth factor

## Abstract

**Highlights:**

**What are the main findings?**

- Norrin ameliorates hyperglycemia-induced neurodegenerative processes leading to RGC apoptosis.
- Norrin attenuates hyperglycemia-induced microvascular leakage and RGC apoptosis by normalizing impaired expression of VEGF and PEDF.

**What are the implications of the main findings?**

- Norrin is important for ameliorating hyperglycemia-induced neurodegeneration in diabetic retinas.
- Our results highlight norrin as a potential therapeutic target for early neurodegenerative changes in diabetic retinopathy.

**Abstract:**

Diabetic retinopathy is increasingly recognized as a neurovascular disorder rather than a purely vascular disease; however, therapeutic strategies targeting retinal neurodegeneration remain limited. In this study, we investigated the protective effects of norrin against hyperglycemia-induced retinal neurodegeneration and elucidated its underlying molecular mechanisms in diabetic mice. We found that retinal neurodegeneration may precede microvascular leakage in diabetic retinas. Norrin, which is expressed in the inner retina, was significantly downregulated under diabetic conditions. Intravitreal supplementation of norrin markedly attenuated hyperglycemia-induced neurodegenerative processes, leading to retinal ganglion cell (RGC) apoptosis, including oxidative stress, inflammation, and neuropathological alterations such as reactive gliosis, glutamate excitotoxicity, and synaptic dysfunction. Norrin also reduced hyperglycemia-induced microvascular leakage and RGC apoptosis by normalizing vascular endothelial growth factor (VEGF) overexpression and restoring pigment epithelium-derived factor (PEDF) levels. Notably, PEDF upregulated by norrin effectively suppressed neurodegenerative processes induced by hyperglycemia or VEGF, thereby preserving RGC function. These findings identify norrin as a critical modulator of hyperglycemia-induced retinal neurodegeneration through restoration of the VEGF–PEDF balance. Our results highlight norrin as a potential therapeutic target for early neurodegenerative changes in diabetic retinopathy.

## 1. Introduction

Diabetic retinopathy (DR), the most common microvascular complication of diabetes, is difficult to diagnose in its early stages due to the absence of noticeable symptoms [1,2,3]. DR is a leading cause of blindness worldwide and can result in serious complications, including permanent vision loss, if left untreated [2]. In the non-proliferative phase of DR, vascular abnormalities (e.g., microaneurysms, vascular leakage, and retinal hemorrhages) result in retinal dysfunction and may progress to the proliferative phase [4,5]. During the proliferative phase, growth factors are secreted, stimulating pathological neovascularization [3,6]. These newly formed vessels are fragile and susceptible to rupture, leading to hemorrhage that can ultimately cause retinal detachment and vision loss [4,5].

DR has historically been recognized as a disease caused by microvascular lesions; extensive research has focused on its underlying mechanisms and therapeutic approaches [3,4]. Recent evidence suggests that DR is a disease of the neurovascular unit, comprising simultaneous neurodegeneration and microvascular abnormalities in the retina [7,8]. The pathogenesis of this disorder involves various glial cells and altered expression of their biomarker proteins. Retinal glial cells, including Müller cells and astrocytes, play essential roles in neurotransmitter modulation, blood–retinal barrier maintenance, and retinal blood flow regulation [1,9]. Under diabetic conditions, dysfunction among these cells results in reactive gliosis, characterized by increased expression of glial fibrillary acidic protein (GFAP) and vimentin, whereas the regulatory functions of glutamine synthetase (GS) and glutamate aspartate transporter (GLAST) are impaired, leading to glutamate toxicity and neuronal cell death [10]. Additionally, downregulation of the synaptic vesicle protein synaptophysin (Syp) contributes to neural circuit dysfunction and neurodegeneration in DR [11]. Müller cell activation also induces overexpression of growth factors, including vascular endothelial growth factor (VEGF), which exacerbates neurotoxicity and impairs neuroprotective mechanisms [12]. These glial cell dysfunctions and disrupted expression of growth factors play critical roles in DR pathogenesis, and represent potential therapeutic targets [9].

Current treatments for DR have primarily targeted microvascular abnormalities, including intravitreal injections of anti-angiogenic agents or steroids; however, these approaches have limited therapeutic efficacy and are associated with various adverse effects [4,13]. Anti-VEGF therapies offer potential benefits in treating ocular neovascular diseases (e.g., diabetic macular edema [DME] and neovascularization) but may lead to serious complications, including endophthalmitis and retinal detachment [1,13]. Moreover, VEGF plays a critical role in neuronal survival: repeated VEGF neutralization may result in retinal neuronal loss [14]. Intravitreal steroids are effective for treating DME but can elevate intraocular pressure, increasing the risk of glaucoma and cataracts [15,16]. There is increasing evidence that neuronal damage may occur prior to vascular abnormalities in DR, highlighting the need for treatments that target neurodegeneration [17,18]. Thus, novel therapeutic strategies addressing neurodegeneration may complement current vasculopathy-based treatments.

Norrin, an atypical signaling protein encoded by the Norrie disease protein (*NDP*) gene, participates in angiogenesis and neuroprotection within retinal and neural tissues by activating the Wnt/β-catenin signaling pathway [19,20]. In the retinas of oxygen-induced retinopathy mice, norrin activates the Wnt/β-catenin signaling pathway, promoting retinal angiogenesis and enhancing the integrity of the blood–retinal barrier [20,21]. In NDP-knockout mice, norrin deficiency delays the development of the superficial retinal vascular plexus, resulting in abnormal vessel formation [22,23]. In addition to its angiogenic function, norrin exhibits neuroprotective effects in the retina [20]. Norrin deficiency leads to extensive reactive gliosis of Müller cells and progressive loss of retinal ganglion cells (RGCs), culminating in substantial disorganization of the ganglion cell layer in NDP-knockout mice [20,23]. Norrin protects RGCs from N-methyl-D-aspartate (NMDA)-induced excitotoxicity by stimulating the production of neurotrophic factors in Müller cells, thereby enhancing RGC axon survival and reducing apoptosis [24,25]. Collectively, these findings suggest that norrin functions as both a vascular regulator and a neuroprotective factor in the retina, making it a promising candidate for novel therapeutic strategies for DR. However, the effects of norrin on diabetes-induced vascular and neuronal dysfunction in the retina remain unclear.

We hypothesized that intravitreal administration of norrin mitigates the hyperglycemia-induced imbalance in VEGF and pigment epithelium-derived factor (PEDF) expression, thereby protecting against neuronal damage in the retinas of diabetic mice. We found that RGC apoptosis, a key indicator of neurodegeneration, may precede microvascular leakage and these pathological symptoms were effectively attenuated by intravitreal supplementation of norrin. Notably, norrin normalized dysregulated VEGF and PEDF expression, reduced oxidative stress and inflammation, and alleviated neuropathological alterations in diabetic retinas. Furthermore, PEDF upregulated by norrin reversed VEGF-induced neuronal damage and RGC functional impairment. These findings suggest that norrin ameliorates hyperglycemia-induced retinal neurodegeneration by restoring the VEGF–PEDF balance and highlight its potential as a neurotrophic therapeutic strategy for diabetic retinopathy.

## 2. Materials and Methods

### 2.1. Generation of Diabetic Mice

Six- or ten-week-old male C57BL/6 mice were obtained from DBL (Eumseong, Korea). Mice were housed in groups of six per filtered-top cage under specific pathogen-free conditions in a temperature-controlled room with a 12 h light/dark cycle. Animals had free access to standard chow and water and were allowed to acclimatize to the housing conditions for at least 4 days prior to experimentation. After acclimatization, mice were randomly assigned to either normal or diabetic groups.

Diabetes was induced by a single intraperitoneal injection of streptozotocin (STZ; 150 mg/kg body weight; MilliporeSigma, Burlington, MA, USA) freshly dissolved in 100 mmol/L citrate buffer (pH 4.5), as previously described [26]. Mice were considered diabetic when fasting glucose levels reached ≥19 mmol/L in the presence of polyuria and glucosuria. Glucose levels and body weight were monitored weekly (*n* = 12). Normal and diabetic mice (*n* = 3 per group) were anesthetized with 3% isoflurane and subjected to analysis of retinal neurodegeneration and microvascular leakage up to 6 weeks after STZ injection.

To evaluate the effects of norrin or PEDF on diabetes-induced microvascular leakage, diabetic mice (*n* = 3 per group) were anesthetized with 3% isoflurane and intravitreally injected with PBS, the indicated amounts of norrin (R&D Systems, Minneapolis, MN, USA), or 100 ng PEDF (Abeomics, San Diego, CA, USA) in 2 μL at 4 weeks after STZ injection. Normal mice (*n* = 3 per group) underwent sham procedures.

To assess the effects of norrin or PEDF on molecular events associated with diabetes-induced neurodegeneration, diabetic mice (*n* = 3 per group) were anesthetized with 3% isoflurane and intravitreally injected with PBS, 100 ng norrin, or 100 ng PEDF in 2 μL once daily for six consecutive days beginning 3 weeks after STZ injection. Normal mice (*n* = 3 per group) underwent sham procedures. Sample sizes were determined based on previous studies using similar experimental designs [27].

All animal procedures were conducted in accordance with the Guide for the Care and Use of Laboratory Animals published by the National Institutes of Health and were approved by the Institutional Animal Care and Use Committee of Kangwon National University (approval No. KW-230227-2). The study was performed and reported in compliance with the ARRIVE guidelines.

### 2.2. Measurement of Vascular Leakage by Fundus Fluorescein Angiography and Fluorescein Isothiocyanate–Dextran Angiography in Mouse Retinas

Vascular leakage was assessed using fundus fluorescein angiography and fluorescein isothiocyanate (FITC)–dextran angiography. For fundus fluorescein angiography, mice were intraperitoneally injected with 10% fluorescein sodium (100 μL/20 g body weight; Alcon, Fort Worth, TX, USA) diluted fivefold with phosphate-buffered saline (PBS). Pupils were dilated with a topical iridodilator (Santen Pharmaceutical, Osaka, Japan), and eye gel (Samil Pharmaceutical, Seoul, Korea) was applied to prevent corneal drying. Fundus images were acquired using the Phoenix MICRON IV system (Phoenix MICRON, Bend, OR, USA), and vascular leakage was quantified (*n* = 6 eyes) by measuring the fluorescence intensity of fluorescein in the captured images.

Vascular leakage was also evaluated by FITC–dextran angiography, as previously described [6,28]. Briefly, after anesthesia with 3% isoflurane, 1.25 mg of 500 kDa FITC–dextran (MilliporeSigma) was injected into the left ventricle and allowed to circulate for 5 min. Eyes were then enucleated, fixed with 4% paraformaldehyde, and retinas were dissected in a Maltese cross configuration. Leakage in the superficial vessels of whole-mount retinas was visualized by confocal microscopy (K1-Fluo; Nanoscope Systems, Daejeon, Korea) and quantified by measuring the fluorescence intensity of FITC-dextran extravasated from retinal vessels, expressed as relative fluorescence intensity normalized to normal control mice (*n* = 6 retinas).

### 2.3. Measurement of Neuro-Apoptosis in Mouse Retinal Sections

Neuro-apoptosis in mouse retinas was assessed using an APO-BrdU TUNEL (terminal deoxynucleotidyl transferase-mediated dUTP–biotin nick-end labeling) assay kit (BD Biosciences, San Jose, CA, USA), as previously described [8]. Briefly, retinal sections (10 μm) were fixed and incubated with a DNA-labeling solution containing terminal deoxynucleotidyl transferase and 5-bromo-2′-deoxyuridine for 1 h at 37 °C. Sections were then incubated with an FITC-labeled 5-bromo-2′-deoxyuridine antibody for 30 min and stained with 1 μg/mL DAPI (MilliporeSigma) for 10 min. Apoptotic cells were visualized using confocal microscopy (K1-Fluo) and quantified by counting the number of TUNEL-positive cells (*n* = 6 retinas).

### 2.4. Immunofluorescence Staining of Mouse Retinal Sections

Protein expression was visualized in retinal sections from normal and diabetic mice by immunofluorescence, as previously described [8]. Briefly, mouse eyes were enucleated and rapidly frozen in Optimal Cutting Temperature compound (Sakura Finetek USA, Torrance, CA, USA). Retinal sections were prepared by cutting unfixed retinal tissues at a thickness of 10 μm using a microtome-cryostat (Leica Biosystems, Wetzlar, Germany). After fixation and permeabilization, the sections were incubated overnight with polyclonal antibodies against norrin (1:200; Novus Biologicals, Centennial, CO, USA), interleukin-17A (IL-17A) (1:200; Invitrogen, Carlsbad, CA, USA), Syp (1:200; Invitrogen), GS (1:200; Invitrogen), and brain-specific homeobox/POU domain protein 3A (Brn3a) (1:200; Abcam, Cambridge, UK). The sections were also incubated with monoclonal antibodies against GFAP (1:200; Cell Signaling Technology, Danvers, MA, USA), interleukin-1β (IL-1β) (1:200; Abcam), tumor necrosis factor-α (TNF-α) (1:200; Cell Signaling Technology), vimentin (1:200; Cell Signaling Technology), GLAST (1:200; Cell Signaling Technology), PEDF (1:200; Abcam), and VEGF (1:200; Invitrogen). The sections were then incubated for 2 h with Alexa Fluor™ 546-conjugated goat anti-rabbit IgG (1:200; Invitrogen). Protein expression was visualized by confocal microscopy and quantitatively analyzed by measuring fluorescence intensity, expressed as relative fluorescence intensity normalized to normal control mice (*n* = 6 retinas).

### 2.5. Immunofluorescence Staining of Whole-Mount Mouse Retinas

Colocalization of GFAP and norrin was visualized in whole-mount retinas by immunofluorescence, as previously described [6]. Briefly, enucleated eyes were fixed with 4% paraformaldehyde, and whole-mount retinas were dissected in a Maltese cross configuration and post-fixed with acetone for 3 min at −20 °C. The retinas were permeabilized with 1.0% Triton X-100, blocked with 2% bovine serum albumin in Tris-buffered saline plus Tween, and incubated with a monoclonal antibody against norrin (1:200; Novus Biologicals). The retinas were then sequentially incubated with an FITC-conjugated goat anti-rabbit IgG (1:200; Invitrogen) for 2 h and an Alexa Fluor™ 647-conjugated GFAP monoclonal antibody (1:200; Invitrogen) for 6 h. Norrin and GFAP expression patterns were co-visualized by confocal microscopy, and norrin expression was quantitatively analyzed by measuring fluorescence intensity, expressed as relative fluorescence intensity normalized to normal control mice (*n* = 6 retinas).

To visualize Brn3a expression, whole-mount retinas were fixed, permeabilized, and incubated overnight at 4 °C with a monoclonal antibody against Brn3a (1:200; Abcam). The retinas were then stained with Alexa Fluor™ 546-conjugated goat anti-rabbit IgG (1:200; Invitrogen). Brn3a expression was visualized in whole-mount retinas using confocal microscopy and quantitatively analyzed (*n* = 6 retinas) by measuring fluorescence intensity and counting the number of Brn3a-positive RGCs.

### 2.6. RNA Extraction, cDNA Synthesis, and Real-Time PCR Analysis of Mouse Retinas

Total RNA was extracted from mouse retinas using TRIzol reagent (Invitrogen). Two isolated retinas were homogenized in 1 mL of TRIzol using a hand homogenizer, mixed with 200 µL of chloroform (MilliporeSigma), and centrifuged at 12,000× *g* for 10 min at 4 °C to separate the phases. The upper aqueous phase containing RNA was transferred to a new tube, mixed with an equal volume of isopropanol (MilliporeSigma), incubated on ice for 10 min, and centrifuged at 12,000× *g* for 10 min at 4 °C. The resulting RNA pellet was washed once with 75% ethanol, air-dried at room temperature, dissolved in 20 µL of DNase/RNase-free water (Invitrogen), and incubated at 50 °C for 5 min to ensure complete dissolution.

Complementary DNA (cDNA) was synthesized from the purified RNA using a commercial cDNA synthesis kit (iNtRON Biotechnology, Seongnam, Korea). Quantitative real-time PCR (qRT-PCR) was performed using a SYBR Green-based qPCR kit (iNtRON Biotechnology) in a final reaction volume of 20 µL. The thermal cycling conditions were as follows: initial denaturation at 95 °C for 10 min, followed by 40 cycles of denaturation at 95 °C for 20 s, annealing at 60 °C for 40 s, and extension at 72 °C for 5 min. The sequences of primer pairs were for murine Norrin 5′-GACGGACTGGCAAGAAAAGAG-3′ (forward) and 5′-TCAGGCACCCAAACCACAAT-3′ (reverse) and for murine β-actin 5′-GCTACAGCTTCACCACCACA-3′ (forward) and 5′-AAGGAAGGCTGGAAAAGAGC-3′ (reverse). Relative mRNA expression levels were quantified using the QuantStudio™ 3 Real-Time PCR System (Thermo Fisher Scientific, Waltham, MA, USA).

### 2.7. Intravitreal Injection of Norrin in Diabetic Mice

After anesthesia induction with 3% isoflurane, mice received intravitreal supplementation of norrin through a single injection [27] with the indicated amounts of recombinant human norrin, or via repeated intravitreal injections of 100 ng recombinant human norrin every 2 days or daily for 6 days (three or six injections in total, respectively). Mice were also administered with VEGF or PEDF through a single intravitreal injection of 100 ng recombinant mouse PEDF or 100 ng human VEGF-165 recombinant protein (Cell Signaling Technology). Subsequently, the mice were subjected to assessments of vascular leakage, neuro-apoptosis, and immunofluorescence.

### 2.8. Enzyme-Linked Immunosorbent Assay Measurements of IL-6, GLAST, and VEGF Levels in Mouse Retinas

IL-6, GLAST, and VEGF expression levels were measured in mouse retinas using a mouse IL-6 ELISA (enzyme-linked immunosorbent assay) kit (R&D Systems), a mouse GLAST ELISA kit (LSBio, Lynnwood, WA, USA), and a mouse VEGF ELISA kit (R&D Systems), respectively, in accordance with the manufacturer’s instructions and as previously described [27]. Retinal lysates were centrifuged at 17,000 × *g* for 15 min at 4 °C, and protein concentrations (*n* = 3, two retinas/experiment) were determined using a microplate spectrophotometer (Epoch; BioTek, Winooski, VT, USA).

### 2.9. Hematoxylin and Eosin Staining of Mouse Retinal Sections

Hematoxylin and eosin (H&E) staining of retinal sections was performed as previously described [6]. Retinal sections (10 μm) were obtained at a distance of approximately 0.5–0.7 mm from the optic nerve head and fixed with 4% paraformaldehyde for 30 min. H&E staining was carried out using Harris hematoxylin solution (MilliporeSigma) for 10 min, followed by 0.2% eosin (MilliporeSigma) for 1 min. Stained sections were examined using confocal microscopy.

### 2.10. Measurements of Reactive Oxygen Species and Superoxide Levels in Mouse Retinal Sections

Reactive oxygen species (ROS) and superoxide levels in mouse retinal sections were measured using CellROX™ Green reagent and dihydroethidium, respectively (Thermo Fisher Scientific), as previously described [6]. Briefly, unfixed retinal sections, prepared using a microtome-cryostat, were incubated at 37 °C with 5 μmol/L CellROX™ Green reagent or dihydroethidium in serum-free M199 medium for 30 min. Stained sections were visualized by confocal microscopy. ROS and superoxide levels were quantified by measuring the fluorescence intensity (*n* = 6 retinas).

### 2.11. Western Blot Analysis

Western Blot analysis was performed as previously described [28]. Protein extracts from retinas were separated by sodium dodecyl sulfate–polyacrylamide gel electrophoresis and then transferred to polyvinylidene fluoride membranes. The membranes were incubated with monoclonal antibodies against vimentin (1:2000; Cell Signaling Technology), GS (1:2000; Invitrogen), and PEDF (1:2000; Abcam) and then incubated with a horseradish peroxidase-conjugated secondary antibody. Protein bands were visualized using a ChemiDoc system (Bio-Rad, Hercules, CA, USA), and protein expression levels were quantified by densitometry and normalized to β-actin expression.

### 2.12. Statistical Analysis

Data were analyzed using OriginPro 2015 software (OriginLab, Northampton, MA, USA) [6]. Data are presented as the mean ± standard deviation (SD) of three or six independent experiments. Statistical significance was determined using one-way analysis of variance (ANOVA) followed by Holm–Sidak’s multiple comparisons test. *p* values < 0.05 were considered statistically significant.

## 3. Results

### 3.1. Neurodegeneration May Precede Microvascular Leakage in the Retinas of Diabetic Mice

**Figure 1:**
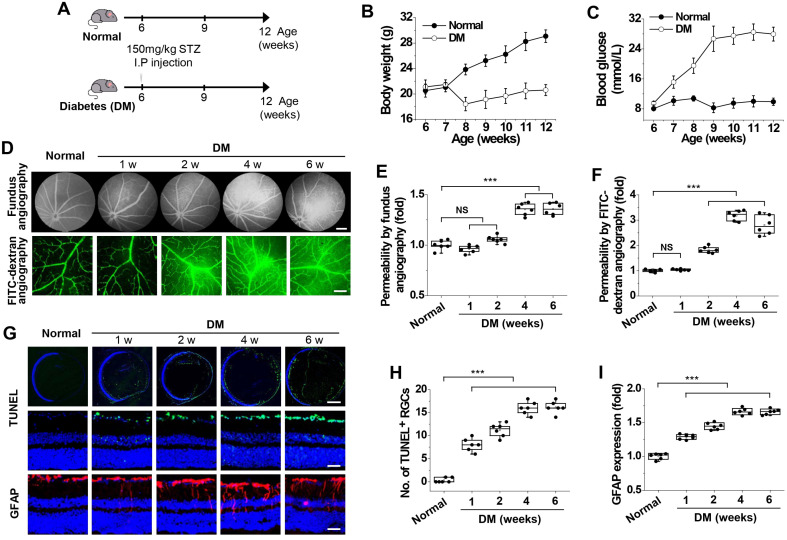
A mouse model for the investigation of hyperglycemia-induced neurodegeneration and vascular leakage in diabetic retinas. Six-week-old C57BL/6 mice were intraperitoneally injected with streptozotocin. Vascular leakage, ganglion cell apoptosis, and glial fibrillary acidic protein (GFAP) expression in the retina were analyzed weekly. (**A**) Schematic illustration of diabetic mouse generation. (**B**,**C**). Body weight (**B**) and blood glucose levels (**C**) were monitored weekly (*n* = 12). (**D**–**F**). Vascular leakage was visualized by fundus fluorescein angiography (scale bar, 300 μm) and FITC-dextran angiography (scale bar, 100 μm) (**D**) and then quantified (*n* = 6) by measuring the fluorescence intensity of fluorescein (**E**) and FITC-dextran (**F**). (**G**). Apoptotic cells were visualized by TUNEL staining (green), with nuclear counterstaining using DAPI (blue) in retinal sections. Images in the middle row (scale bar, 100 μm) are magnified views of the top row images (scale bar, 600 μm). GFAP expression was visualized by immunofluorescence (scale bar, 100 μm). (**H**). Quantification of TUNEL-positive retinal ganglion cells (TUNEL^+^ RGCs) (*n* = 6). (**I**). Quantification of GFAP expression by measuring fluorescence intensity (*n* = 6). Statistical significance was determined using one-way ANOVA with Holm–Sidak’s multiple comparisons test. NS, non-significant; *** *p* < 0.001.

To investigate hyperglycemia-induced pathological characteristics in DR, we generated diabetic mice via STZ injection (Figure 1A) and then examined microvascular leakage and apoptosis in diabetic retinas. Compared with non-diabetic mice, diabetic mice exhibited sustained weight loss and hyperglycemia for up to 6 weeks after STZ injection (Figure 1B,C). Microvascular leakage was assessed by fundus fluorescein angiography and FITC–dextran angiography. Fundus fluorescein angiography revealed substantial microvascular permeability at 4 weeks after diabetes induction (Figure 1D,E), whereas FITC–dextran angiography of whole-mount retinas detected microvascular leakage as early as 2 weeks (Figure 1D,F), indicating that microvascular leakage began 2 weeks post induction.

To assess hyperglycemia-induced retinal neurodegeneration, we analyzed RGC apoptosis and GFAP expression in diabetic mice. RGC apoptosis, a hallmark of retinal neurodegeneration, was clearly observed at 1 week; the number of apoptotic RGCs increased over time after diabetes induction (Figure 1G,H). Glial cell activation was assessed through the upregulation of GFAP expression, a biomarker of reactive gliosis and key pathological feature of neurodegeneration in DR [8]. Similar to RGC apoptosis, GFAP expression was detected at 1 week and progressively increased until 6 weeks (Figure 1G,I). These findings suggest that neurodegeneration may occur prior to microvascular leakage in the retinas of diabetic mice.

### 3.2. Optimized Intravitreal Injection of Norrin Ameliorates Hyperglycemia-Induced Vascular Leakage and RGC Apoptosis Without Cytotoxicity in the Retinas of Diabetic Mice

**Figure 2:**
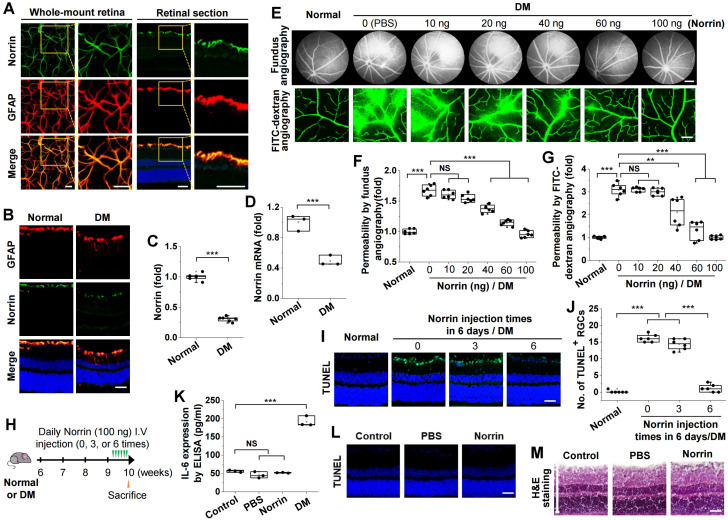
Optimized intravitreal norrin injection inhibits hyperglycemia-induced retinal vascular leakage and RGC apoptosis in diabetic mice. (**A**). Colocalization of norrin (green) and GFAP (red) in whole-mount retinas and retinal sections. Enlarged images of the boxed regions in the left panels are shown in the right panels. Scale bar, 100 μm. (**B**,**C**). Norrin expression (green) was visualized by confocal microscopy with GFAP (red) co-staining in retinal sections from normal and diabetic mice (DM) (**B**) and quantified by measuring fluorescence intensity (*n* = 6) (**C**). Scale bar, 100 μm. (**D**). Expression of norrin mRNA in the retinas (*n* = 3, two retinas/experiment). (**E**–**G**). Diabetic mice received intravitreal injections (2 μL) of PBS or the indicated amounts of norrin. One day after injection, vascular leakage was visualized by fundus fluorescein angiography (scale bar, 300 μm) and FITC-dextran angiography (scale bar, 100 μm) (**E**) and then quantified by measuring fluorescence intensity (*n* = 6) (**F**,**G**). (**H**). Schematic of intravitreal injections (0, 3, or 6 times over 6 days) of PBS or 100 ng norrin into normal or diabetic mice before sacrifice. (**I**,**J**). TUNEL-positive cells (green) were visualized by confocal microscopy with nuclear counterstaining using DAPI (blue) in retinal sections, according to the frequency of norrin administration (**I**), and quantified in the ganglion cell layer (*n* = 6) (**J**). Scale bar, 100 μm. (**K**–**M**). No cytotoxicity was observed in normal mice intravitreally injected six times over 6 days with PBS or 100 ng norrin. (**K**). ELISA determined no change in interleukin-6 (IL-6) levels in retinal lysates (*n* = 3, two retinas/experiment). (**L**). No apoptosis was detected by TUNEL staining (green) in retinal sections. Scale bar, 100 μm. (**M**). No histopathological changes were observed by H&E staining in retinal sections. Scale bar, 100 μm. Statistical significance was determined using one-way ANOVA with Holm–Sidak’s multiple comparisons test. NS, non-significant; ** *p* < 0.01; *** *p* < 0.001.

To investigate the beneficial effect of norrin on DR, we first examined norrin expression in the retinas of C57BL/6 mice. Norrin colocalized with GFAP in whole-mount retinas. In retinal sections, colocalized norrin and GFAP signals were predominantly observed in the inner retinal region adjacent to the ganglion cell layer, indicating that norrin expression is mainly associated with GFAP-positive glial cells within the inner retina (Figure 2A). Compared with normal mice, expression levels of norrin protein and mRNA were substantially reduced in diabetic mice (Figure 2B–D).

We optimized the intravitreal injection protocol for norrin to evaluate its protective effects against hyperglycemia-induced microvascular leakage and RGC apoptosis. Various doses of norrin were administered to diabetic mice to assess its inhibitory effect on microvascular leakage. Fundus fluorescein angiography showed that norrin attenuated microvascular leakage in a dose-dependent manner; maximal efficacy was observed at 100 ng (Figure 2E,F). Similar results were obtained via FITC-dextran angiography (Figure 2E,G). We then used TUNEL assays to explore the optimal injection frequency of norrin for reducing RGC apoptosis. Hyperglycemia-induced RGC apoptosis was inhibited by daily intravitreal injection of 100 ng norrin for 6 consecutive days, but not by injection every 2 days over the same period (Figure 2H–J). However, daily injection of 100 ng norrin for 6 consecutive days did not induce inflammation, RGC apoptosis, or pathological changes in the retinas of normal mice (Figure 2K–M). These results suggest that norrin, secreted by glial cells, is downregulated in the retinas of diabetic mice; optimized intravitreal injection of norrin ameliorates hyperglycemia-induced microvascular leakage and RGC apoptosis without inducing cytotoxicity.

### 3.3. Norrin Inhibits Hyperglycemia-Induced Oxidative Stress, Inflammation, and Neuropathological Alterations Such as Reactive Gliosis, Glutamate Excitotoxicity, and Synaptic Dysfunction in the Retinas of Diabetic Mice

**Figure 3:**
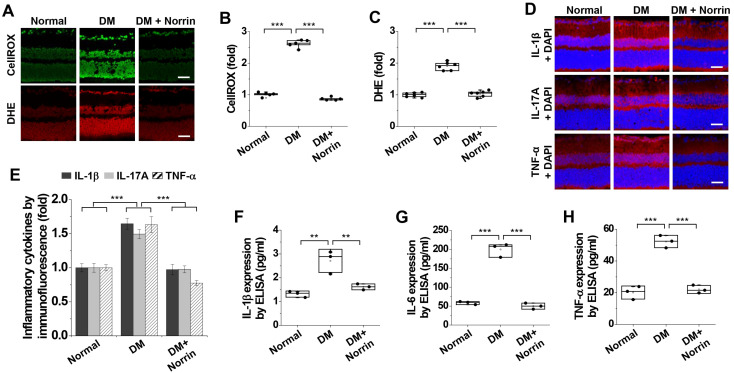
Norrin inhibits hyperglycemia-induced oxidative stress and inflammation in the retinas of diabetic mice. Diabetic mice received intravitreal injections (2 μL) of PBS (DM) or 100 ng norrin (DM + Norrin) in 2 μL for six consecutive days beginning 3 weeks after STZ injection and subjected to analyses of reactive oxygen species (ROS) and superoxide generation, as well as the expression of inflammatory cytokines. (**A**–**C**). ROS and superoxide generation were visualized using CellROX™ Green (green) and dihydroethidium (DHE) (red), respectively, in retinal sections (**A**) and then quantified by measuring fluorescence intensity (*n* = 6) (**B**,**C**). Scale bar, 100 μm. (**D**,**E**). Expression patterns of interleukin-1β (IL-1β, interleukin-17A (IL-17A), and tumor necrosis factor-α (TNF-α) were visualized by immunofluorescence (red) in retinal sections (**D**) and quantified by measuring fluorescence intensity (*n* = 6) (**E**). Scale bar, 100 μm. (**F**–**H**). Quantification of retinal IL-1β (**F**), IL-6 (**G**), and TNF-α (**H**) levels by ELISA (*n* = 3, two retinas/experiment). Statistical significance was determined using one-way ANOVA with Holm–Sidak’s multiple comparisons test. ** *p* < 0.01; *** *p* < 0.001.

To investigate the pathological mechanisms by which norrin ameliorates hyperglycemia-induced neurodegeneration, we examined its inhibitory effects on oxidative stress and inflammation in the retinas of diabetic mice. Compared with non-diabetic controls, diabetic mice exhibited increased ROS and superoxide generation, which was suppressed by intravitreal injection of norrin (Figure 3A–C). Norrin also effectively inhibited the upregulated expression of inflammatory cytokines—including IL-1β, IL-17A, TNF-α, and IL-6—in diabetic retinas (Figure 3D–H).

**Figure 4:**
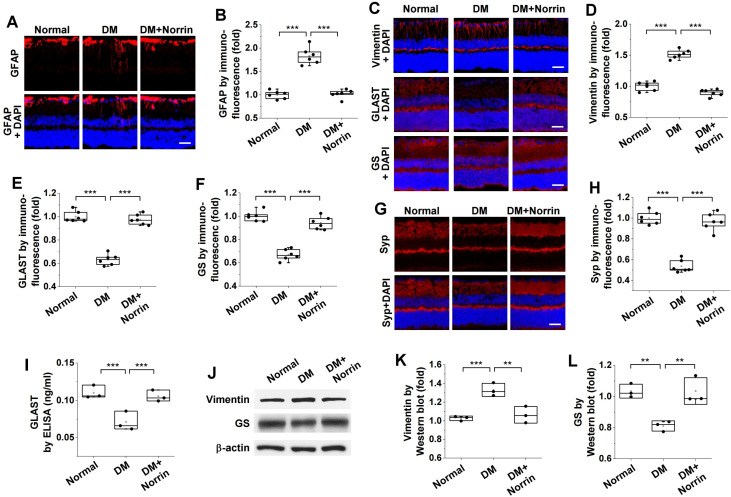
Norrin inhibits hyperglycemia-induced neurodegeneration in the retinas of diabetic mice. Diabetic mice received intravitreal injections (2 μL) of PBS or 100 ng norrin and subjected to analyses of neurodegeneration biomarkers in the retina. (**A**,**B**). GFAP expression (red) was visualized by immunofluorescence in retinal sections (**A**) and quantified by measuring fluorescence intensity (*n* = 6) (**B**). Scale bar, 100 μm. (**C**–**F**). Expression patterns (red) of vimentin, glutamate aspartate transporter (GLAST), and glutamine synthetase (GS) were visualized by immunofluorescence in retinal sections (**C**) and then quantified by measuring fluorescence intensity (*n* = 6) (**D**–**F**). Scale bar, 100 μm. (**G**,**H**). Synaptophysin (Syp) expression (red) was visualized by immunofluorescence in retinal sections (**G**) and quantified by measuring fluorescence intensity (*n* = 6) (**H**). Scale bar, 100 μm. (**I**). GLAST levels were analyzed by ELISA in retinal lysates (*n* = 3, two retinas/experiment). (**J**–**L**). Expression patterns of vimentin and GS were analyzed by Western Blot in retinal lysates (**J**) and quantified by densitometry (*n* = 3, two retinas/experiment) (**K**,**L**). Statistical significance was determined using one-way ANOVA with Holm–Sidak’s multiple comparisons test. ** *p* <0.01; *** *p* < 0.001.

Next, we evaluated the protective effect of norrin on hyperglycemia-induced neuropathological alterations by using immunofluorescence, ELISA, and Western Blot analyses to measure the expression levels of biomarker proteins associated with reactive gliosis, glutamate excitotoxicity, and synaptic dysfunction. Hyperglycemia induced overexpression of GFAP and vimentin, markers of reactive gliosis; this overexpression was inhibited by intravitreal injection of norrin (Figure 4A–D). The inhibitory effect of norrin on vimentin overexpression was further confirmed by Western Blot analysis (Figure 4J,K). Norrin reversed the hyperglycemia-induced downregulation of GLAST and GS (Figure 4C,E,F); its protective effect against glutamate toxicity was supported by ELISA and Western Blot analyses (Figure 4I,J,L). Additionally, norrin ameliorated synaptic dysfunction by restoring the expression of Syp, which had been downregulated by hyperglycemia (Figure 4G,H). Taken together, these findings indicate that norrin attenuates hyperglycemia-induced neurodegenerative processes, including oxidative stress, inflammation, and neuropathological alterations, such as reactive gliosis, glutamate excitotoxicity, and synaptic dysfunction, in the retinas of diabetic mice.

### 3.4. Norrin Ameliorates Hyperglycemia-Induced Microvascular Leakage and RGC Apoptosis by Normalizing VEGF Overexpression and PEDF Downregulation in the Retinas of Diabetic Mice

**Figure 5:**
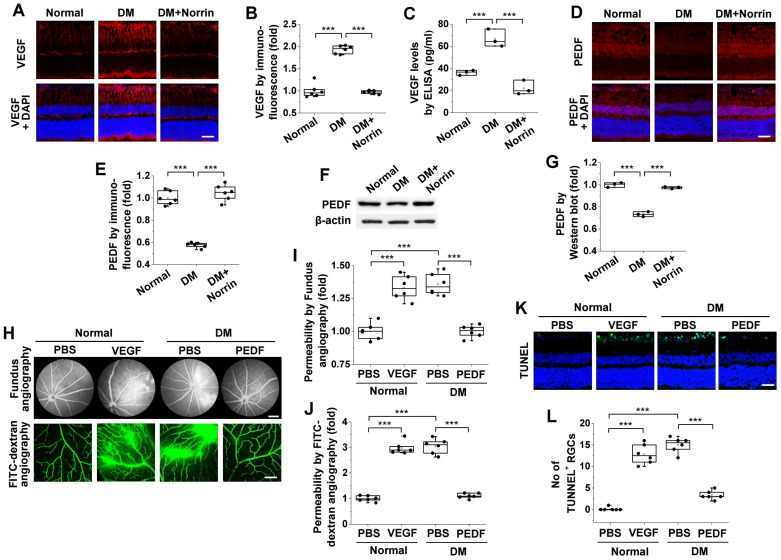
Norrin inhibits hyperglycemia-induced retinal vascular leakage and RGC apoptosis by modulating VEGF and PEDF. (**A**–**G**). Diabetic mice received intravitreal injections (2 μL) of PBS or 100 ng norrin and subjected to analyses of pigment epithelium-derived factor (PEDF) and vascular endothelial growth factor (VEGF) expression patterns, vascular leakage, and RGC apoptosis in the retina. (**A**,**B**). VEGF expression (red) was visualized by immunofluorescence in retinal sections (**A**) and quantified by measuring fluorescence intensity (*n* = 6) (**B**). Scale bar, 100 μm. (**C**). Quantification of VEGF expression levels by ELISA in retinal lysates (*n* = 3, two retinas/experiment). (**D**–**G**). PEDF expression was analyzed by immunofluorescence (**D**,**E**) and Western Blot (**F**,**G**). (**D**,**E**). PEDF expression was visualized by immunofluorescence in retinal sections (**D**) and quantified by measuring fluorescence intensity (*n* = 6) (**E**). Scale bar, 100 μm. (**F**,**G**). PEDF expression was analyzed by Western Blot in retinal lysates (**F**) and quantified by densitometry (*n* = 3, two retinas/experiment) (**G**). (**H**–**J**). Vascular leakage was visualized by fundus fluorescein angiography (scale bar, 300 μm) and by FITC–dextran angiography in whole-mount retinas (scale bar, 100 μm) (**H**) and then quantified by measuring fluorescence intensity (*n* = 6) (**I**,**J**). (**K**,**L**). Normal or diabetic mice were treated with PBS or 100 ng PEDF by daily intravitreal injection for 6 consecutive days (see Figure 2G). Apoptotic cell death was visualized by TUNEL staining (green) in retinal sections (**K**), and TUNEL-positive cells were quantified in the RGC layer (*n* = 6) (**L**). Scale bar, 100 μm. Statistical significance was determined using one-way ANOVA with Holm–Sidak’s multiple comparisons test. *** *p* < 0.001.

To investigate the molecular mechanism by which norrin attenuates hyperglycemia-induced microvascular leakage and RGC apoptosis, we examined its effects on VEGF and PEDF expression patterns in the retinas of diabetic mice. Hyperglycemia elevated VEGF expression, and this overexpression was suppressed by norrin administration (Figure 5A–C). Conversely, hyperglycemia reduced the expression of the neuroprotective factor PEDF; this reduction was reversed by norrin supplementation (Figure 5D–G). These results demonstrate that norrin normalizes VEGF overexpression and PEDF downregulation in diabetic retinas.

Next, we evaluated the effects of intravitreally injected VEGF and PEDF on microvascular leakage and RGC apoptosis in the retinas of normal and diabetic mice. VEGF induced microvascular leakage in the retinas of normal mice, whereas PEDF suppressed hyperglycemia-induced microvascular leakage in diabetic retinas (Figure 5H–J). VEGF also induced RGC apoptosis in normal mice; however, PEDF inhibited hyperglycemia-induced RGC apoptosis in diabetic mice (Figure 5K,L). Taken together, these findings suggest that impaired expression of VEGF and PEDF plays a crucial role in the pathogenesis of DR. Furthermore, norrin ameliorates these pathological conditions in the retinas of diabetic mice.

### 3.5. PEDF Ameliorates Hyperglycemia- or VEGF-Induced Oxidative Stress, Inflammation, and Neuropathological Alterations in the Retinas of Diabetic Mice

**Figure 6:**
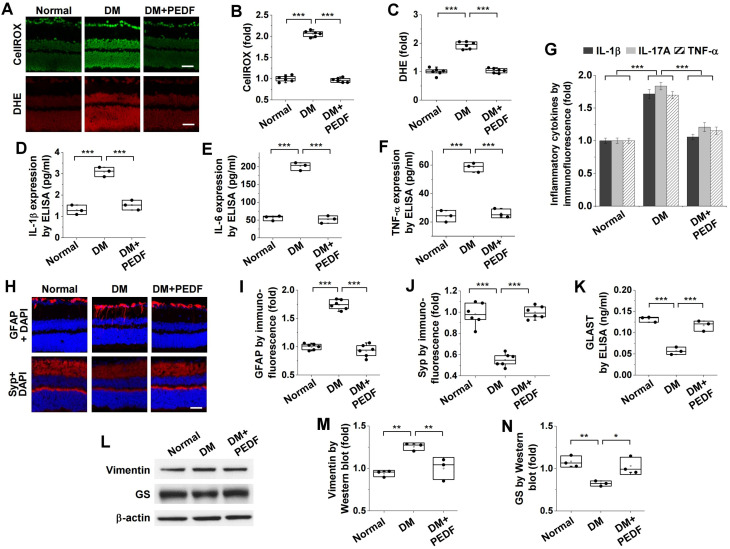
PEDF inhibits hyperglycemia-induced oxidative stress, inflammation, and neurodegeneration in the retinas of diabetic mice. Diabetic mice received intravitreal injections (2 μL) of PBS or 100 ng PEDF, and subjected to analyses of ROS and superoxide generation (**A**–**C**), expression of inflammatory cytokines (**D**–**G**), and neurodegeneration biomarkers (**H**–**N**) in the retina. (**A**–**C**). ROS and superoxide generation were visualized using CellROX™ Green (green) and DHE (red), respectively, in retinal sections (**A**) and then quantified by measuring fluorescence intensity (*n* = 6) (**B**,**C**). Scale bar, 100 μm. (**D**–**F**). Expression levels of IL-1β (**D**), IL-6 (**E**), and TNF-α (**F**) were determined by ELISA (*n* = 3, two retinas/experiment). (**G**). Expression patterns of IL-1β, IL-17A, and TNF-α were determined by immunofluorescence (*n* = 6). H–J. Expression patterns of GFAP and Syp were visualized by immunofluorescence (**H**) and quantified by measuring fluorescence intensity (*n* = 6) (**I**,**J**). Scale bar, 100 μm. (**K**). Quantification of GLAST expression levels by ELISA (*n* = 3, two retinas/experiment). (**L**–**N**). Expression levels of vimentin and GS were analyzed by Western Blot (**L**) and quantified by densitometry (*n* = 3, two retinas/experiment) (**M**,**N**). Statistical significance was determined using one-way ANOVA with Holm–Sidak’s multiple comparisons test. * *p* < 0.05; ** *p* < 0.01; *** *p* < 0.001.

We investigated the inhibitory effects of PEDF, whose expression was restored by norrin (Figure 5D–G), on hyperglycemia-induced oxidative stress, inflammation, and neuropathological alterations in the retinas of diabetic mice. Intravitreal administration of PEDF suppressed hyperglycemia-induced ROS and superoxide generation in diabetic retinas (Figure 6A–C). PEDF also inhibited the hyperglycemia-induced expression of inflammatory cytokines, including IL-1β, IL-17A, TNF-α, and IL-6 (Figure 6D–G). Moreover, PEDF attenuated the overexpression of GFAP (Figure 6H,I) and vimentin (Figure 6L,M), whereas it mitigated the downregulation of Syp (Figure 6H,J), GLAST (Figure 6K), and GS (Figure 6L,N) in diabetic retinas. These results demonstrate that PEDF supplementation effectively ameliorates the neurodegenerative processes leading to hyperglycemia-induced RGC apoptosis in the retinas of diabetic mice.

**Figure 7:**
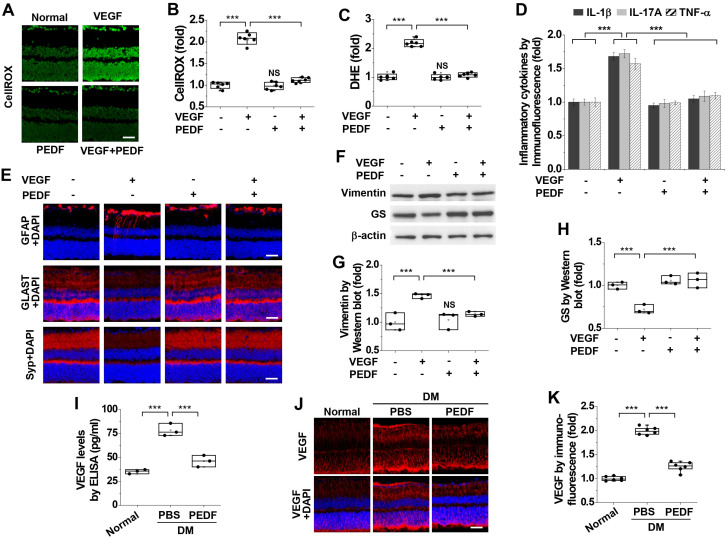
PEDF inhibits VEGF-induced oxidative stress, inflammation, and neurodegeneration in the retinas of diabetic mice. (**A**–**H**). Non-diabetic C57BL/6 mice (10 weeks old) received intravitreal injections (2 μL) of PBS, 100 ng VEGF, 100 ng PEDF, or both VEGF and PEDF for 24 h and then subjected to analyses of ROS and superoxide generation, inflammation, and neurodegeneration in the retina. (**A**,**B**). ROS generation was visualized using CellROX™ Green (**A**) and quantified by measuring fluorescence intensity (*n* = 6) (**B**). Scale bar, 100 μm. (**C**). Superoxide generation was measured using DHE (*n* = 6). (**D**.) Expression patterns of IL-1β, IL-17A, and TNF-α were determined by immunofluorescence (*n* = 6). (**E**). Expression patterns of GFAP, GLAST, and Syp were visualized by immunofluorescence. Scale bar, 100 μm. (**F**–**H**). Expression levels of vimentin and GS were analyzed by Western Blot (**F**) and quantified by densitometry (*n* = 3, two retinas/experiment) (**G**,**H**). (**I**–**K**). Diabetic mice were intravitreally injected with PBS or 100 ng PEDF and subjected to analysis of VEGF expression in the retina. (**I**). Quantification of VEGF expression levels by ELISA (*n* = 3, two retinas/experiment). (**J**,**K**). VEGF expression was visualized by immunofluorescence in retinal sections (**J**) and quantified by measuring fluorescence intensity (*n* = 6) (**K**). Scale bar, 100 μm. Statistical significance was determined using one-way ANOVA with Holm–Sidak’s multiple comparisons test. NS, non-significant; *** *p* < 0.001.

Given that VEGF levels were elevated in the retinas of diabetic mice (Figure 5A–C), we investigated the beneficial effects of PEDF on VEGF-induced oxidative stress, inflammation, and neuropathological alterations by intravitreal co-administration of VEGF and PEDF in the retinas of normal mice. PEDF suppressed VEGF-induced ROS and superoxide generation (Figure 7A–C). PEDF also inhibited VEGF-induced expression of inflammatory cytokines, including IL-1β, IL-17A, and TNF-α (Figure 7D). Additionally, PEDF normalized the VEGF-induced overexpression of GFAP (Figure 7E) and vimentin (Figure 7F,G), as well as the downregulation of GLAST (Figure 7E), Syp (Figure 7E), and GS (Figure 7F,H). No changes in neurodegenerative processes were observed in normal retinas treated with PEDF alone. Furthermore, PEDF attenuated hyperglycemia-induced elevation of VEGF levels in diabetic retinas (Figure 7I–K), indicating that PEDF is involved in the inhibition of VEGF expression. Taken together, these results demonstrate that PEDF ameliorates oxidative stress, inflammation, and neuropathological alterations induced by hyperglycemia or VEGF in the retinas of diabetic mice.

### 3.6. Norrin Ameliorates Hyperglycemia-Induced Functional Impairment of RGCs in the Retinas of Diabetic Mice

**Figure 8:**
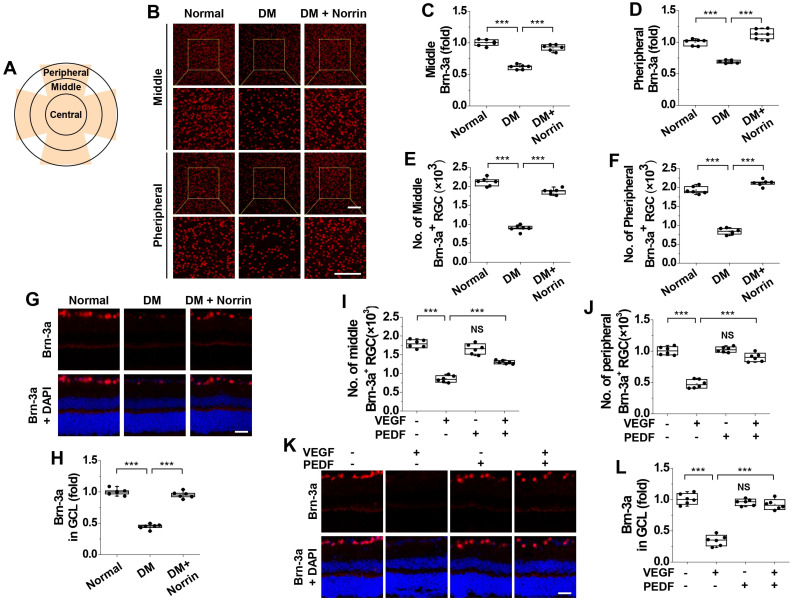
Norrin ameliorates hyperglycemia-induced functional impairment of RGCs through PEDF in the retinas of diabetic mice. (**A**–**H**). Diabetic mice received intravitreal injections (2 μL) of PBS or 100 ng norrin and then subjected to analysis of Brn3a expression by confocal microscopy in whole-mount retinas (**B**–**F**) and retinal sections (**G**,**H**). (**A**). A schematic diagram showing three regions of the ganglion cell layer in a whole-mount retina. (**B**–**D**). Brn3a expression was visualized by confocal microscopy in the middle and peripheral regions (**B**) and then quantified by measuring fluorescence intensity (*n* = 6) (**C**,**D**). Scale bar, 100 μm. (**E**,**F**). Quantification of Brn3a-positive RGCs in the middle (**E**) and peripheral (**F**) regions (*n* = 6). (**G**,**H**). Brn3a expression was visualized by immunofluorescence in retinal sections (**G**) and quantified by measuring fluorescence intensity (*n* = 6) (**H**). Scale bar, 100 μm. (**I**–**L**). Non-diabetic C57BL/6 mice (10 weeks old) were intravitreally injected with PBS, 100 ng VEGF, 100 ng PEDF, or both VEGF and PEDF and subjected to analysis of Brn3a expression by immunofluorescence in whole-mount retinas (**I**,**J**) and retinal sections (**K**,**L**). (**I**,**J**). Quantification of Brn3a-positive RGCs in the middle (**I**) and peripheral (**J**) regions (*n* = 6). (**K**,**L**). Brn3a expression was visualized by confocal microscopy in retinal sections (**K**) and quantified by measuring fluorescence intensity (*n* = 6) (**L**). Scale bar, 100 μm. Statistical significance was determined using one-way ANOVA with Holm–Sidak’s multiple comparisons test. NS, non-significant; *** *p* < 0.001.

Subsequently, we investigated the neuroprotective effect of norrin by using Brn3a immunofluorescence to evaluate functional recovery among impaired RGCs in the retinas of diabetic mice (Figure 8A). Hyperglycemia reduced Brn3a expression in the middle and peripheral regions of whole-mount retinas; this reduction was reversed by norrin supplementation (Figure 8B–D). Norrin also reversed hyperglycemia-induced decreases in Brn3a-positive cell numbers in the middle and peripheral regions of diabetic retinas (Figure 8E,F). Consistent with these observations, norrin restored Brn3a expression in the RGC layer, which had been reduced by hyperglycemia (Figure 8G,H).

The beneficial effects of norrin on RGC functional recovery were also examined through intravitreal co-administration of VEGF and PEDF in normal mice. VEGF reduced the numbers of Brn3a-positive cells in the middle and peripheral regions of normal retinas; these numbers were restored by PEDF (Figure 8I,J). Additionally, PEDF reversed the VEGF-induced decrease in Brn3a expression in the RGC layer (Figure 8K,L). Consistent with these observations, VEGF-induced decreases in Brn3a expression were reversed by PEDF in the middle and peripheral regions of whole-mount retinas. Our findings indicate that norrin reverses hyperglycemia-induced RGC functional impairment through PEDF in the retinas of diabetic mice.

Overall, norrin ameliorates hyperglycemia-induced RGC apoptosis and functional impairment by restoring the balance between VEGF and PEDF expression, thereby reducing oxidative stress, inflammation, and neuropathological alterations, including reactive gliosis, glutamate excitotoxicity, and synaptic dysfunction, in the retinas of diabetic mice.

## 4. Discussion

DR, the most common microvascular complication of diabetes, remains a leading cause of blindness in developed countries [3,4]. DR has historically been recognized as a microvascular disease; however, increasing evidence supports the notion that DR is a disorder of the neurovascular unit, involving both microvascular dysfunction and neurodegeneration [1,8,17]. Current treatments primarily target vascular abnormalities but are inadequate for addressing neurodegeneration [13,15]. Thus, the identification of new therapeutic strategies targeting early retinal neurodegeneration is essential for the effective treatment of DR [8]. In the present study, we found that norrin was associated with reduced hyperglycemia-induced RGC apoptosis, an early hallmark of retinal neurodegeneration that may precede microvascular leakage, possibly through modulation of VEGF overexpression and PEDF downregulation in the retinas of diabetic mice. Norrin expression was downregulated in diabetic retinas, and intravitreal supplementation of norrin was associated with attenuation of neurodegenerative processes linked to RGC functional impairment and apoptosis, including oxidative stress, inflammation, neuropathological alterations such as reactive gliosis, glutamate excitotoxicity, and synaptic dysfunction. PEDF, a well-established neuroprotective factor, was found to attenuate VEGF-induced neurodegenerative processes associated with RGC apoptosis. Thus, norrin may represent a potential therapeutic candidate for DR by targeting early stages of retinal neurodegeneration.

Retinal neurodegeneration may occur earlier than microvascular leakage in the retinas of diabetic mice. Recent observations suggest that retinal neurodegeneration occurs at early stages and may be followed by microvascular dysfunction during the pathogenesis of DR [1,18,29]. Clinical and experimental studies have revealed inner retinal neurodegeneration in the absence of microvascular changes in diabetic patients and diabetic mouse models [18]. Retinal neurodegeneration is a potential early event in DR and may contribute to the development of microvascular abnormalities [8,29]. In the present study, we demonstrated that RGC apoptosis and glial cell activation were initiated at 1 week after diabetes induction, whereas microvascular leakage was observed at 2 weeks. These findings suggest that retinal neurodegeneration may occur prior to vasculopathy in the pathogenesis of DR, emphasizing the importance of developing therapeutic approaches that target early-stage retinal neurodegeneration for effective treatment.

Treatment strategies for vision-threatening DR include laser photocoagulation, intravitreal injections of anti-angiogenic agents or steroids, and vitreoretinal surgery; these methods primarily target microvascular complications during the advanced stages of DR [4,8]. Pan-retinal photocoagulation is effective in reducing the rate of vision loss in eyes with DME or proliferative DR; however, it is associated with adverse effects such as reduced night and color vision [4,30]. Intravitreal injections of anti-VEGF agents demonstrate greater efficacy in reducing vision loss among patients with DME and inhibiting retinal neovascularization among those with proliferative DR, but they have not achieved consistent benefits in the majority of DR patients [1,4,15]. Intravitreal steroids have become important in DME treatment but are limited by frequent ocular side effects, including cataracts and glaucoma [4,15]. Vitreoretinal surgery is utilized for the management of proliferative DR complicated by non-clearing vitreous hemorrhage or tractional retinal detachment; it typically results in limited visual improvement [31]. Thus, current treatments targeting microvascular abnormalities during the late stages of DR are associated with limited efficacy and adverse effects. Efforts to overcome these limitations require new therapies for DR that focus on early stages of retinal neurodegeneration.

Norrin, a growth factor encoded by the *NDP* gene, is primarily secreted by glial cells (e.g., Müller cells and astrocytes). It plays a crucial role in retinal vascular formation and development by activating the Wnt/β-catenin signaling pathway [21,24]. Norrin attenuates VEGF-induced vascular permeability through the interaction of disheveled-1 with claudin-5 in rat retinas [19,32]. Additionally, as a neurotrophic factor, norrin supports neuronal survival and differentiation, promotes the expression of fibroblast growth factor-2 to suppress neuroinflammation, and stabilizes the extracellular environment [20,25]. Through the Wnt/β-catenin pathway, norrin increases the expression of neuroprotective factors such as leukemia inhibitory factor and endothelin-2, thereby exerting protective effects against NMDA-induced excitotoxic damage to RGCs [24,25,33]. Due to these neuroprotective properties, norrin may represent a therapeutic candidate for DR; however, its protective effects against diabetes-induced retinal neurodegeneration remains unknown.

In the present study, we found that norrin was downregulated in the retina of diabetic mice. Intravitreal supplementation of norrin attenuated hyperglycemia-induced reactive gliosis and RGC apoptosis, which were observed as early as 1 week after diabetes induction, potentially by normalizing dysregulated VEGF and PEDF expression. Moreover, norrin inhibited neurodegenerative processes associated with RGC apoptosis, including oxidative stress, inflammation, neuropathological alterations, and RGC functional impairment. These findings support a protective effect of norrin and suggest its role in preserving retinal neurons during the early stages of DR. Furthermore, norrin demonstrated beneficial effects on retinal microvascular damage, indicating its potential as a therapeutic agent targeting both retinal neurodegeneration and microvascular dysfunction in early DR. However, norrin did not significantly alter the expression levels of Frizzled-4, LRP5/6, and β-catenin in the retinas of STZ-induced diabetic mice, suggesting that its effects on VEGF and PEDF expression may occur via a pathway independent of the canonical β-catenin cascade.

An imbalance in VEGF and PEDF expression may play a critical role in the pathogenesis of DR. VEGF, a potent angiogenic and vascular permeability factor, is essential for normal retinal function [34,35]. However, excessive VEGF expression leads to pathological neovascularization, oxidative stress, and inflammatory responses, ultimately resulting in retinal neuronal damage [6,8,34]. By contrast, PEDF acts as a potent neuroprotective and anti-angiogenic factor, maintaining microvascular homeostasis in the retina [3,36]. Clinical studies have identified elevated VEGF levels and reduced PEDF levels in the retinas of patients with proliferative DR [36,37]. A hyperglycemia-induced imbalance between VEGF and PEDF has been associated with neovascularization in the retinas of proliferative DR mice [6]. In this study, hyperglycemia-induced VEGF overexpression and PEDF downregulation were associated with microvascular leakage and RGC apoptosis. Norrin attenuated both microvascular leakage and neurodegenerative processes associated with RGC apoptosis by restoring the balance of VEGF and PEDF expression. Intravitreal supplementation of PEDF effectively attenuated VEGF-induced neurodegenerative processes (e.g., oxidative stress, inflammation, neuropathological alterations, and RGC functional impairment) in the retinas of diabetic mice. These findings suggest that impaired VEGF and PEDF expression patterns may contribute to DR pathogenesis and highlight norrin as a potential therapeutic agent for DR. 

Several limitations warrant consideration. First, while our study provides consistent molecular and histological evidence supporting the protective effects of norrin on retinal pathology, future studies incorporating functional assays will be important to further validate its therapeutic potential in improving retinal function. Second, although our findings suggest that PEDF contributes to the protective effects of norrin, the lack of loss-of-function or inhibition experiments limits our ability to establish a direct causal relationship; therefore, future studies employing PEDF neutralization or genetic knockdown approaches will be necessary to confirm its mechanistic role. Third, although repeated daily intravitreal injections were effective in this experimental setting, such a dosing regimen is not clinically feasible; therefore, future studies are warranted to evaluate more clinically relevant strategies, including reduced injection frequency or sustained delivery approaches. Fourth, in this study, in vivo experiments were conducted using six retinas from three mice per treatment group to minimize animal use. Future studies using more animals (e.g., six mice per group) would enhance statistical robustness and strengthen the validity of the findings. Finally, our mechanistic conclusions are based on an STZ-induced diabetic mouse model; therefore, validation in additional preclinical models and human retinal tissues will be required to enhance translational relevance.

## 5. Conclusions

Our study demonstrates that norrin ameliorates hyperglycemia-induced retinal neurodegeneration, which may precede microvascular leakage, by normalizing VEGF overexpression and restoring PEDF levels in the retinas of diabetic mice. Supplementation with PEDF effectively attenuated VEGF-induced neurodegenerative processes. Collectively, these results identify norrin as a promising therapeutic candidate for targeting early retinal neurodegeneration in diabetic retinopathy, potentially overcoming the limitations of current treatments that primarily focus on vasculopathy.

## Data Availability

The original contributions presented in this study are included in the article Further inquiries can be directed to the corresponding author.

## Abbreviations

ANOVA	analysis of variance
Brn3a	brain-specific homeobox/POU domain protein 3A
DME	diabetic macular edema
DR	diabetic retinopathy
ELISA	enzyme-linked immunosorbent assay
FITC	fluorescein isothiocyanate
GFAP	glial fibrillary acidic protein
GLAST	glutamate aspartate transporter
GS	glutamine synthetase
IL-17A	interleukin-17A
IL-1β	interleukin-1 β
NDP	Norrie disease protein
NMDA	N-methyl-D-aspartate
PEDF	pigment epithelium-derived factor
RGC	retinal ganglion cell
Syp	synaptophysin
TNF-α	tumor necrosis factor-α
TUNEL	terminal deoxynucleotidyl transferase-mediated dUTP-biotin nick-end labeling
VEGF	vascular endothelial growth factor
